# Supplementary material for: Cloud BioLinux: pre-configured and on-demand bioinformatics computing for the genomics community
Source: BMC Bioinformatics. 2012 Mar 19;13:42. doi: 10.1186/1471-2105-13-42 (PMC3372431; doi:10.1186/1471-2105-13-42)
Supplement: Additional file 1 — Supplementary 1 Cloud BioLinux software documentation in the form of a mini, self-contained website. Users need to download and uncompress the .zip file, and open through a web browser the "index.html" file available on the main directory. (ZIP 1823 kb). [file 1471-2105-13-42-S1.ZIP › Cloud-BioLinux-Package-Documentation/docs/Phyml.html]

Bio-Linux Software Documentation Pages

Back to search form

## Phyml

|  |  |
| --- | --- |
| Name | Phyml |
| Description | PhyML is a phylogeny software based on the maximum-likelihood princ iple. Early PhyML versions used a fast algorithm to perform Nearest Neighbor Interchanges (NNIs), in order to improve a reasonable starting tree topology. Since the original publication (Guindon and Gascuel 2003), PhyML has been widely used (>1,250 citations in ISI Web of Science), due to its simplicity and a fair accuracy/speed compromise. In the mean time research around PhyML has continued. We designed an efficient algorithm to search the tree space using Subtree Pruning and Regrafting (SPR) topological moves (Hordijk and Gascuel 2005), and proposed a fast branch test based on an approximate likelihood ratio test (Anisimova and Gascuel 2006). However, these novelties were not included in the official version of PhyML, and we found that improvements were still needed in order to make them effective in some practical cases. PhyML 3.0 achieves this task. It implements new algorithms to search the space of tree topologies with user-defined intensity. A non-parametric, Shimodaira-Hasegawa-like branch test is also available. The program provides a number of new evolutionary models and its interface was entirely re-designed. We tested PhyML 3.0 on a large collection of real data sets to ensure that the new version is stable, ready-to-use and still reasonably fast and accurate. |
| Homepage | http://www.atgc-montpellier.fr/phyml/usersguide.php |
| Remote Documentation | http://www.atgc-montpellier.fr/phyml/faq.php |
